# High-Dimensional Small-Sample Feature Selection Using Co-Evolutionary Ant Colony Optimization Inspired by Heterosis

**DOI:** 10.3390/biomimetics11060404

**Published:** 2026-06-08

**Authors:** Chunli Xiang, Jing Zhou, Zhiwei Ye, Zenggang Xiong, An Song, Dingfeng Song, Jie Sun

**Affiliations:** 1 School of Computer Science and Artificial Intelligence, Hubei University of Technology, No. 28 Nanli Road, Hongshan District, Wuhan 430068, China; xiangchunli@hbut.edu.cn (C.X.); zhoujing2024@hbut.edu.cn (J.Z.); 102311321@hbut.edu.cn (A.S.); 102413010@hbut.edu.cn (D.S.); sunjie@hbut.edu.cn (J.S.); 2 Hubei Provincial Key Laboratory of Green Intelligent Computing Power Network, No. 28 Nanli Road, Hongshan District, Wuhan 430068, China; 3 Hubei Key Laboratory of Digital Finance Innovation, Hubei University of Economics, No. 8 Yangqiaohu Road, Jiangxia District, Wuhan 430205, China; 4 School of Computer and Information Science, Hubei Engineering University, Xiaogan 432000, China; xzg@hbeu.edu.cn

**Keywords:** high-dimensional feature selection, small sample, ant colony optimization algorithm, self-attention mechanism, hybrid rice optimization algorithm

## Abstract

High-dimensional small-sample data are widely encountered in medical diagnosis, bioinformatics, and industrial inspection, where traditional feature selection methods often suffer from premature convergence and local optima. To address these issues, this paper proposes a Hybrid Breeding-based Co-evolutionary Ant Colony Optimization method (HBACO) for feature selection. Inspired by the principle of hybrid breeding, in which individuals with distinct traits produce superior offspring through cross recombination, inheritance of desirable genes and continuous evolution, the proposed algorithm establishes a three-population collaborative framework. It consists of an ACO-based search population, an HRO-based evolutionary population and a cooperative feedback population that evolve iteratively together. Furthermore, we devise a heuristic strategy integrating correlation and genetic characteristics to help mine high-value feature subsets. Meanwhile, a collaborative pheromone updating mechanism is adopted to realize efficient knowledge sharing among populations. Experiments conducted on 13 high-dimensional datasets, including Colon and Lung, demonstrate that HBACO achieves superior classification accuracy, feature reduction performance, and convergence behavior compared with 10 representative algorithms. Specifically, HBACO improves the average classification accuracy by 3.9% and achieves an average feature dimensionality reduction rate of 91.4%. Statistical tests further confirm the significance of the proposed method. The results indicate that HBACO provides an effective and robust solution for high-dimensional feature selection problems.

## 1. Introduction

With the rapid development of big data and artificial intelligence, high-dimensional data widely exists in computer vision [1], financial early warning [2], industrial detection [3], medical diagnosis [4] and other fields. Such data usually exhibits the typical characteristics of high dimensionality and small sample size, which easily leads to model overfitting and significantly increases the computational complexity of model training. In the biomedical field, such as gene microarray data, due to the high cost and limited quantity of samples, the feature dimension is much larger than the sample size, which brings severe challenges to traditional machine learning methods.

To alleviate the curse of dimensionality, dimensionality reduction techniques have been widely used, mainly including feature extraction and feature selection. Feature extraction methods such as principal component analysis [5], linear discriminant analysis [6] and canonical correlation analysis [7] represent raw data by constructing a new low-dimensional feature space, but they often destroy the physical meaning of original features. In contrast, feature selection directly screens the subset with the strongest discriminative ability from the original feature set. While maintaining data interpretability, it effectively reduces model complexity and is more suitable for practical application scenarios.

Essentially, feature selection is a typical combinatorial optimization problem, whose key lies in designing an efficient search strategy to find the optimal feature subset in an exponential search space. Although traditional deterministic search methods can obtain the global optimal solution, their computational complexity is too high and they are only suitable for low-dimensional problems [8]. Heuristic search methods such as sequential forward/backward search [9] and their improved variants reduce computational cost, but easily fall into local optimum. In recent years, swarm intelligence optimization algorithms have become the mainstream methods to solve high-dimensional feature selection problems due to their excellent global search ability and robustness.

The Ant Colony Optimization algorithm (ACO) is a typical swarm intelligence algorithm, which achieves global search through the positive feedback mechanism of pheromone and exhibits strong optimization ability in combinatorial optimization problems, so it is widely used in feature selection tasks. However, traditional ACO still has obvious limitations in high-dimensional feature selection: its heuristic information design is insufficient, and the search process tends to be a single direction, resulting in premature convergence; meanwhile, the generated feature subsets have high redundancy, which affects the dimensionality reduction effect and classification performance.

However, the recently proposed Hybrid Rice Optimization algorithm (HRO) shows promising performance in balancing global exploration and local exploitation by simulating the diverse search mechanism in the process of hybrid breeding. However, the precise search ability of a single HRO in a discrete high-dimensional search space still needs to be improved. Most existing hybrid swarm intelligence algorithms integrate different optimization strategies merely in a serial or nested manner. They lack in-depth collaboration and dynamic information interaction among diverse populations, which keeps the search and evolution processes relatively independent. Consequently, these algorithms fail to fully exploit the complementary advantages of heterogeneous optimization mechanisms.

Against this background, aiming at the feature selection requirements of high-dimensional small-sample data, how to combine the advantages of ACO in combinatorial optimization and the advantages of HRO in diversity maintenance to construct an efficient and stable feature selection method is still a problem worthy of in-depth study.

The main contributions of this study are summarized as follows:

- An innovative three-population dynamic collaborative structure composed of an ACO-led search group, an HRO-led evolution group, and a collaborative feedback group is proposed.
- A dynamic heuristic mechanism integrating feature correlation and generational inheritance information is designed, which enhances the algorithm’s ability to identify potential key features.
- A dual-population linkage pheromone updating strategy is proposed to realize information sharing among populations.
- Experiments are carried out on 13 high-dimensional benchmark datasets and compared with a variety of classic and state-of-the-art feature selection algorithms. The results show that HBACO achieves remarkable advantages in classification accuracy, feature reduction capability and convergence performance.

The remainder of this paper is organized as follows: Section 2 reviews recent related work. Section 3 introduces the proposed HBACO for feature selection. Experimental results and discussions are provided in Section 4. Finally, Section 5 concludes the paper and outlines potential directions for future research.

## 2. Related Works

Feature selection methods are generally categorized into three types according to the coupling degree with the learner: filter, wrapper, and embedded methods. Filter methods evaluate features independently based on statistical indicators, such as distance metric [10], information theory metric [11], correlation metric [12], and consistency metric [13]. They have high computational efficiency but ignore the interdependencies among features. Wrapper methods evaluate the performance of feature subsets by incorporating specific learners, such as *K*-nearest neighbor, neural network [14], and support vector machine [15]. They can capture the interactive information among features but incur relatively high computational cost. Embedded methods perform feature selection during the model training process, such as $L_{1}$ regularization and random forest. They achieve a trade-off between efficiency and performance, yet the results rely heavily on the specific model structure.

With the emergence of high-dimensional and complex data, traditional feature selection methods still exhibit shortcomings in modeling nonlinear relationships and searching efficiency, which motivates researchers to explore more effective search strategies.

### 2.1. Swarm Intelligence for Feature Selection

Swarm intelligence optimization algorithms achieve efficient global optimization in high-dimensional search spaces by simulating natural population behaviors. Typical algorithms include particle swarm optimization (PSO) [16], grey wolf optimizer (GWO) [17], cuckoo search algorithm [18], and so on. To address the discrete property of feature selection, researchers have proposed various binary encoding strategies to enable these algorithms to adapt to feature subset search tasks.

To alleviate the premature convergence issue of conventional algorithms, numerous improved approaches have been developed. For instance, Ma et al. [19] introduced chaotic initialization and adaptive parameter adjustment mechanisms to enhance search diversity; Gabbi Reddy et al. [20] strengthened the initialization process of PSO using fuzzy logic; Aly et al. [21] integrated the advantages of different algorithms to construct hybrid optimization models for balancing exploration and exploitation.

In addition, multi-objective optimization methods have become a research hotspot. Liu et al. [22] integrated binary grey wolf optimization with cuckoo search for hybrid feature selection, achieving balanced exploration–exploitation via cooperative operators.; Sezgin et al. [23] systematically analyzed the adaptability of different algorithms in multi-objective scenarios; Cai et al. [24] improved the diversity of solution sets via dynamic niche technology.

In recent years, hybrid optimization algorithms have attracted extensive attention. Ye et al. [25] combined HRO and GWO to propose a multi-strategy cooperative optimization method, which enhances population diversity through neighborhood search and crossover operations; Sancar et al. [26] and Chen et al. [27] also improved the performance and stability of feature selection by fusing strategies from different algorithms, respectively.

### 2.2. ACO-Based Feature Selection

As one of the classic swarm intelligence algorithms, ACO has been widely applied to feature selection since its proposal [28]. It guides the search direction through a pheromone update mechanism and delivers competitive performance in combinatorial optimization problems.

To overcome the drawbacks of ACO in high-dimensional problems, various improved strategies have been presented. For example, Paniri et al. [29] utilized maximum mutual information to initialize pheromones for improving search efficiency; Wang et al. [30,31] introduced symmetric uncertainty to measure feature correlation and redundancy; Kakarash et al. [32] integrated graph clustering to strengthen feature evaluation. Furthermore, Karimi et al. [33] and Paniri [34] introduced reinforcement learning to realize dynamic adjustment of heuristic information, thereby enhancing the global search ability of ACO.

In recent years, the combination of ACO and deep learning has emerged as a new research direction. For instance, Xia et al. [35] employed ACO to optimize the performance of deep neural networks; Ma et al. [36] enhanced heuristic information via deep reinforcement learning; Agarwal et al. [37] and Sarhan et al. [38] integrated convolutional neural networks to improve classification performance, respectively. Research on ACO-based high-dimensional feature selection has achieved remarkable progress. Although SAMACO (improved ACO algorithm based on self-attention mechanism) improves the quality of heuristic information through self-attention-based feature correlation modeling and demonstrates competitive feature selection performance [39], its optimization process is still primarily driven by a single-population ACO framework. As a result, the algorithm may suffer from limited population diversity and insufficient information interaction during the later stages of evolution, which can increase the risk of premature convergence and local optima in high-dimensional search spaces. Furthermore, the heuristic information in SAMACO mainly focuses on feature relationships, while the evolutionary inheritance of high-quality search experience is not explicitly exploited.

On the other hand, HRO and its co-evolutionary idea have exhibited excellent optimization performance in recent years across feature selection, federated learning, and complex optimization problems. Relevant studies have demonstrated that HRO can effectively enhance population diversity and global search capability by simulating the co-evolution mechanism in the hybrid breeding process. For instance, HBOFFS improves search performance and convergence efficiency in federated feature selection by integrating immune evolution mechanisms with Cauchy distribution sampling strategies [40]. IMUFFS constructs a cooperative particle swarm optimization framework inspired by the co-evolution principle of HRO, realizing global search and feature collaborative optimization in multi-view federated feature selection [41]. Moreover, CHBSI adopts a three-subpopulation co-evolution mechanism combined with particle swarm optimization, which significantly boosts convergence speed and classification performance in high-dimensional feature selection tasks [42]. These studies verify that the co-evolution and multi-population interaction mechanisms of HRO possess distinct advantages in maintaining population diversity, avoiding local optima, and strengthening global exploration ability.

Although existing hybrid swarm intelligence algorithms improve the performance of feature selection to a certain extent by integrating multiple optimization strategies, most of them adopt serial or loosely coupled structures. These methods lack in-depth collaboration and dynamic information interaction between different optimization mechanisms. As a result, each sub-algorithm performs search and update independently, making it difficult to fully utilize the complementary strengths of heterogeneous optimization mechanisms.

In fact, ACO and HRO exhibit distinct complementary characteristics in optimization behavior. Guided by pheromones, ACO possesses strong local exploitation capability and positive feedback reinforcement mechanism, which can effectively mine potential high-quality feature subsets. Inspired by the breeding process of three-line hybrid rice, HRO maintains population diversity via co-evolution strategies including three-line division, hybridization, self-crossing and resetting, and delivers superior global exploration ability and local optimum escape capability. Hence, the two algorithms are naturally complementary in exploration and exploitation.

As summarized in Table 1, existing swarm-intelligence-based feature selection methods have achieved encouraging results through various biologically inspired search mechanisms. However, most existing approaches rely on a single-population evolutionary structure, which limits information exchange and diversity maintenance during the optimization process. Although SAMACO improves heuristic guidance by incorporating self-attention-based feature correlation modeling, it still lacks an explicit collaborative evolutionary mechanism for exploiting complementary search behaviors and evolutionary inheritance.

Based on the above analysis, this work does not simply realize modular superposition of ACO and HRO. Instead, drawing on the idea of synergistic enrichment of superior traits and intergenerational inheritance in hybrid breeding, we establish a closed-loop collaborative framework integrating search, evolution and feedback. Within this framework, the hereditary enhancement mechanism of HRO acts not only on population evolution, but also reshapes the heuristic information construction and pheromone collaborative update strategies of ACO. On one hand, by combining feature correlation and generational inheritance increment, the heuristic guidance of superior feature combinations is dynamically strengthened. On the other hand, the dual-population linked pheromone update enables cross-population sharing and collaborative transmission of search experience and evolutionary knowledge. This design achieves a better balance between global exploration and local exploitation for high-dimensional feature selection tasks.

## 3. The Proposed Method

**Figure 1:**
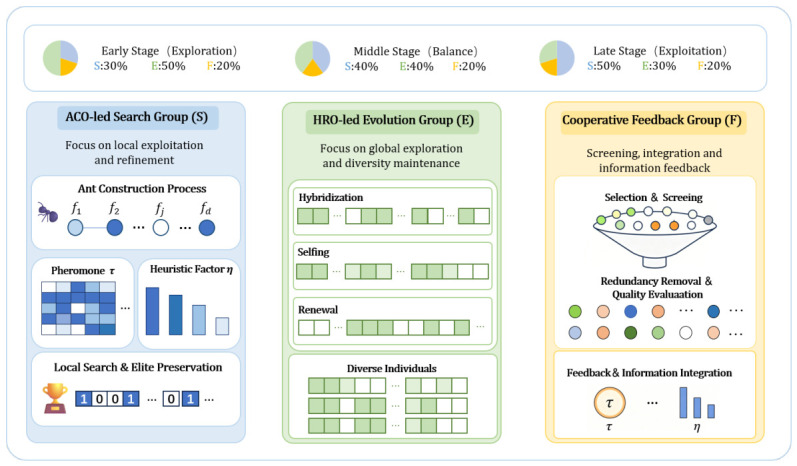
The overall framework of the proposed HBACO method.

In this section, a high-dimensional small-sample feature selection method is proposed based on a co-evolutionary ant colony optimization algorithm with a hybrid breeding mechanism. By integrating the strong optimization ability of ant colony optimization and the dominant trait inheritance idea of hybrid breeding, this method constructs a three-population dynamic collaborative structure, thereby improving the ability of the algorithm to explore excellent feature subsets in high-dimensional feature spaces. The overall framework of the proposed HBACO method is illustrated in Figure 1.

### 3.1. Three-Population Dynamic Cooperative Structure

Most traditional hybrid algorithms take one algorithm as a supplement to the other, lacking deep interaction between them. Inspired by the cooperative breeding mechanism of the three-line hybrid rice breeding system, we propose a co-evolutionary ant colony optimization algorithm based on the hybrid breeding mechanism. It adopts a three-population dynamic cooperative structure consisting of an ACO-dominated search group, an HRO-dominated evolution group, and a collaborative feedback group. Each population undertakes a task corresponding to a component in the hybrid breeding process, and continuous individual exchange and information transmission are conducted among the three populations.

The ACO-dominated search group (corresponding to the maintainer line in HRO) performs feature search based on feature correlation and pheromone concentration, focusing on local optimal regions to mine high-quality feature subsets and inherit the advantages of parental individuals. The HRO-dominated evolution group (corresponding to the restorer line and sterile line in HRO) generates a series of individuals with diverse characteristics through operations such as hybridization, selfing, and renewal, which prevents the search from falling into local optima, provides new search directions for the search group, and increases the genetic diversity of the population. The collaborative feedback group (corresponding to the screening and purification module in HRO) selects excellent individuals from the above two groups. After eliminating redundant individuals, it feeds back the information of dominant traits to both the search group and the evolution group to guide the subsequent search and evolution, and selects superior individuals from hybrid offspring.

This three-population dynamic cooperative structure dynamically adjusts the population size proportion after each iteration according to the fitness distribution. In the early stage, the proportion of the evolution group is increased to 50%, the search group is reduced to 30%, and the feedback group is set to 20%, mainly to expand the search space and enhance population diversity. In the middle stage, the ratio of the three populations is adjusted to 4:4:2 to balance exploration and exploitation. In the late stage, the proportion of the search group is increased to 50%, the evolution group is decreased to 30%, and the feedback group remains 20%, focusing on extracting and refining high-quality feature subsets.

### 3.2. ACO-Led Search Group

To address the problems of slow search speed and premature convergence to local optima in high-dimensional spaces, we introduce an adaptive parameter θ combined with the roulette wheel selection strategy to control the selection process. The probability $p_{i}$ of selecting each feature is determined by the pheromone concentration $\tau_{i}$ and the heuristic information $\eta_{i}$, as shown in Equation (1):

(1) $$p_{i}=\frac{\tau_{i}^{\alpha }\eta_{i}^{\beta }}{\sum_{j=1}^{d}\tau_{j}^{\alpha }\eta_{j}^{\beta }}$$

where $p_{i}$ denotes the selection probability of the *i*-th feature, $\tau_{i}$ is the pheromone concentration of the *i*-th feature, and $\eta_{i}$ represents the heuristic information of the *i*-th feature, which is calculated based on the attention weight scores. α and β are the influence factors of pheromone concentration and heuristic factor, respectively, used to adjust their impact on the probability distribution. *d* is the total number of features.

In high-dimensional feature selection tasks, accurately evaluating the importance of each feature is a crucial step. Traditional feature scoring methods typically assign scores based on statistical metrics or weights derived from machine learning models, yet they ignore correlations and complex interactions among features. The self-attention mechanism was first widely applied and achieved great success in natural language processing. Essentially, it determines the importance of each feature by capturing relationships between features. Accordingly, we adopt the self-attention mechanism to implement feature importance scoring. First, the feature matrix *X* is input into the self-attention module, and three vectors *Q*, *K*, and *V* are obtained through linear transformations. The self-attention weight matrix *A* is then calculated using Equation (2):

(2) $$A=\mathrm{softmax}\left(\frac{QK^{T}}{\sqrt{d_{k}}}\right)$$

where $d_{k}$ is the dimension of the key matrix *K*. The softmax function converts the similarity scores into a probability distribution such that the weights of all features sum to 1. Finally, the attention weight matrix *A* is multiplied by the value matrix *V* to obtain the feature importance score matrix *S*, as shown in Equation (3):

(3) S=AV

Each element in matrix *S* is the importance score of a feature, and a higher score indicates that the feature is more important in this dataset. Next, the average value of each row in matrix *S* is computed to obtain a d×1 feature importance vector. The softmax function is then applied to map the probability values to the range [0,1], yielding the basic heuristic factor η for ACO, as shown in Equation (4):

(4) $$\eta =\mathrm{softmax}\left(\frac{1}{k}\sum_{k=1}^{k}S_{d,k}\right)$$

The probabilities of all features are mapped to segments in the interval [0,1] to form a cumulative probability vector *P*, where each element represents the cumulative probability of the corresponding feature. The adaptive parameter θ is used to determine the number of selection iterations, thereby controlling the size of the selected feature subset. The number of iterations is set to the product of the feature count *d* and θ, i.e., N=θ×d. The parameter θ is adaptively adjusted according to the feature dimension *d*, as shown in Equation (5):

(5) $$\theta =\left\{\begin{array}{cc} \theta_{min}+\frac{\theta_{max}-\theta_{min}}{1+e^{-\lambda (d-d_{0})}} & d>d_{0}\\ \theta_{min} & \mathrm{otherwise} \end{array}\right.$$

where $\theta_{min}$ and $\theta_{max}$ are the minimum and maximum values of θ, respectively, λ is a tuning parameter, and $d_{0}$ is the threshold of feature dimension.

Different from the fixed heuristic factor in traditional algorithms, HBACO designs a dynamic heuristic factor that integrates feature correlation and generational inheritance increment. Based on the feature correlation calculated by the self-attention mechanism, it superimposes the weight of high-quality feature combinations emerging during the iteration, enabling the algorithm to accurately capture potential feature associations. The specific formula is shown in Equation (6):

(6) $$\eta_{i}\left(t\right)=\eta_{base,i}+\Delta \eta_{i}\left(t\right)$$

where $\eta_{base,i}$ is the basic heuristic factor of feature *i*, which is obtained by normalizing the feature correlation calculated by the self-attention mechanism; $\Delta \eta_{i}\left(t\right)$ is the generational inheritance increment at the *t*-th iteration, which is mainly used to enhance the heuristic information for features with consistently good performance. If feature *i* appears in the elite individuals of the collaborative feedback group for two consecutive iterations, $\Delta \eta_{i}\left(t\right)$ increases; if it does not appear for three consecutive iterations, $\Delta \eta_{i}\left(t\right)$ decreases. The value range of $\Delta \eta_{i}\left(t\right)$ is limited to [0,0.5] to prevent search solidification caused by excessively large heuristic factors.

### 3.3. HRO-Led Evolution Group

By simulating the biological mechanisms of synergistic enrichment of superior traits and intergenerational genetic enhancement in the three-line hybrid rice breeding process, HRO realizes the continuous accumulation and co-evolution of high-quality solutions. In three-line hybrid rice breeding, the maintainer line, restorer line and sterile line continuously strengthen desirable traits and gradually eliminate inferior genes through hybridization, selfing, screening and resetting, so as to breed new varieties with stronger adaptability. During the initial stage, all individuals are sorted according to their fitness values, forming the population: $X=\{X_{1},X_{2},\ldots ,X_{n}\}$ where *n* represents the population size. The maintainer line, which consists of individuals with the highest fitness values, is defined as: $M=\left\{X_{1},X_{2},X_{3},\ldots ,X_{m}\right\},m=\left\lfloor n/3\right\rfloor$. The sterile line is composed of individuals exhibiting the poorest fitness $X_{s}=\left\{X_{2m+1},X_{2m+2},\ldots ,X_{n}\right\},$ while the remaining individuals make up the restorer line $X_{r}=\left\{X_{m},X_{m+1},\ldots ,X_{2m}\right\}$.

Hybridization: The sterile individuals are updated during this step. The process of generating new individuals through hybridization is given by Equation (7),

(7) $$\begin{array}{c} X_{s(i)}\left(t+1\right)=k_{1}\cdot X_{s(j)}\left(t\right)+\left(1-k_{1}\right)\cdot X_{m}\left(t\right), \end{array}$$

where $X_{s(i)}\left(t+1\right)$ represents a new sterile-line individual generated in iteration *t*, $X_{m}\left(t\right)$ and $X_{s(j)}\left(t\right)$ represent individuals randomly drawn from the maintainer and sterile lines, respectively. While $k_{1}$ denotes a uniformly distributed random variable within [0,1], the subsequent parameters $k_{2}$ and $k_{3}$ share the same distribution and are utilized in later stages of the algorithm.

Selfing: In this step, the individuals in the restorer line integrate genetic information from other individuals to facilitate the evolution of the subpopulation towards an optimal solution. This process is formulated as Equation (8),

(8) $$\begin{array}{c} X_{r(i)}\left(t+1\right)=k_{2}\cdot \left(X_{\mathrm{best}}\left(t\right)-X_{r(j)}\left(t\right)\right)+X_{r(i)}\left(t\right). \end{array}$$

In this equation, $X_{r(i)}\left(t+1\right)$ refers to a newly generated individual obtained through self-fertilization between two restorer individuals *i* and *j*. The element $X_{\mathrm{best}}\left(t\right)$ represents the current best individual in iteration *t*, whereas $X_{r(j)}\left(t\right)$ represents another restorer individual randomly chosen from the population.

Renewal: If a restorer-line individual fails to update for a predefined number of consecutive iterations ($SC_{\max}$), the algorithm resets it by randomly selecting new values from the search space. This mechanism is described as Equation (9),

(9) $$\begin{array}{c} X_{r(i)}\left(t+1\right)=k_{3}\cdot \left(V_{\max}-V_{\min}\right)+X_{r(i)}\left(t\right)+V_{\min}, \end{array}$$

where $X_{r(i)}\left(t\right)$ represents the restorer individual that has not been updated, and $V_{\max}$ and $V_{\min}$ are the maximum and minimum values of the search space.

### 3.4. Cooperative Feedback Group

Pheromone update is the core link driving the search direction and population evolution of ant colony algorithms. However, a single update mode relying only on local search information is difficult to balance convergence accuracy and population diversity in high-dimensional spaces. Therefore, this paper constructs a pheromone linkage update mechanism based on cooperative task sharing between two populations in HBACO, enabling the search group and the evolution group to realize knowledge sharing and collaboration through pheromone distribution. The formula for the dual-population linkage pheromone update is shown in Equation (10):

(10) $$\begin{array}{ccc} \tau_{i}\left(t+1\right)=\left(1-\rho \right)\cdot \tau_{i}\left(t\right) & +\Delta \tau_{aco,i}\left(t\right) & +\Delta \tau_{hro,i}\left(t\right) \end{array}$$

where ρ is the dynamic evaporation coefficient; $\Delta \tau_{aco,i}\left(t\right)$ is the pheromone increment from the ACO search group, which is calculated by Equations (11) and (12):

(11) $$\Delta \tau_{aco,i}\left(t\right)=\gamma \times \frac{C}{f_{k}}$$

(12) $$\gamma =\gamma_{min}+\frac{\gamma_{max}-\gamma_{min}}{1+e^{-\lambda (t-t_{0})}}$$

where *C* is a constant, usually set to 1, $f_{k}$ is the fitness value of the *k*-th elite ant, γ is a weight factor used to control the intensity of pheromone update, $\gamma_{min}$ and $\gamma_{max}$ are the minimum and maximum values of γ, respectively, λ is a tuning parameter, *t* is the current iteration number, and $t_{0}$ is the threshold of the iteration number. $\Delta \tau_{hro,i}\left(t\right)$ is the pheromone feedback increment from the HRO evolution group. If the fitness of a feature combination in the evolution group improves by more than 10% compared with the previous iteration, $\Delta \tau_{hro,i}\left(t\right)$ for all features in this combination is increased by 0.05; otherwise, it is set to 0. The calculation method of the pheromone feedback increment from the evolution group is shown in Equation (13):

(13) $$\Delta \tau_{hro,i}\left(t\right)=\left\{\begin{array}{cc} 0.05, & \mathrm{if}\;\frac{F_{hro}\left(t\right)-F_{hro}\left(t-1\right)}{F_{hro}\left(t-1\right)}>10\%\\ 0, & \mathrm{otherwise} \end{array}\right.$$

where $F_{hro}\left(t\right)$ represents the fitness value of the target feature combination in the evolution group at the *t*-th iteration; $F_{hro}\left(t-1\right)$ represents the fitness value of the same feature combination at the (t−1)-th iteration. The relative improvement threshold of the feature combination’s fitness is set to 10%. Small improvements below the threshold are generally considered random fluctuations with no practical optimization value. Only when the improvement exceeds 10% is $\Delta \tau_{hro,i}\left(t\right)$ assigned to 0.05 for all features in the combination, so that the evolving population can feed back the excellent results. Otherwise, it is set to zero to avoid unnecessary pheromone accumulation.

### 3.5. Computational Complexity Analysis

Let *N* be the population size, *D* the feature dimension, and *T* the maximum number of iterations. Before iteration, HBACO constructs the self-attention correlation matrix with a time complexity of $O(D^{2})$ and initializes three collaborative populations at the cost of O(ND). In each iteration, the path construction and heuristic guidance of the ACO search group, the three-line grouping as well as hybridization, selfing and renewal operators of the HRO evolution group, and the dual-population pheromone update together with generational inheritance increment adjustment of the collaborative feedback group all operate at the complexity of O(D) or O(ND). Consequently, the total time complexity of HBACO is derived as $O(D^{2}+TND)$. $O(D^{2})$ denotes the one-time preprocessing overhead, and O(TND) is the dominant iterative term that increases linearly with *T*, *N* and *D*. The collaborative feedback mechanism only introduces extra computation of O(TD), which does not alter the asymptotic order with respect to *N*, *T* and *D*. This demonstrates that the proposed three-population collaborative framework achieves closed-loop coordination among search, evolution and feedback with acceptable overhead. The space complexity of HBACO is $O(D^{2}+ND)$. Specifically, $O(D^{2})$ is used to store the self-attention correlation matrix, and O(ND) is allocated for the solutions of the three populations.

Notably, the additional modules introduced in HBACO improve search efficiency through information sharing rather than expanding the search space or adopting nested optimization. Therefore, HBACO maintains the same asymptotic complexity order as traditional ACO and evolutionary algorithms for feature selection. It reveals that the performance improvement in HBACO stems from efficient exploitation of search information instead of extra computational consumption, which achieves a good balance between optimization performance and computational cost.

### 3.6. Fitness Function

In the feature selection process, the design of the objective function plays a decisive role in finding the optimal feature subset by the optimization method. The objective function adopted in this paper considers both classification accuracy and feature subset size. On the premise of ensuring high classification accuracy, it selects as few features as possible to simplify the model, save computation time, and reduce model complexity and computational cost. The objective function is calculated as shown in Equation (14):

(14) $$\mathrm{Fitness}=\lambda \cdot \left(1-\mathrm{Accuracy}\right)+\mu \cdot \frac{n}{N}$$

where Accuracy is used to measure the performance of the classifier; a larger value indicates better classification performance. In the objective function Fitness, λ and μ are weight coefficients that control the trade-off between classification accuracy and feature selection ratio, satisfying λ∈[0,1] and λ+μ=1. *n* denotes the size of the selected feature subset, and *N* is the total number of features. In our experiments, the parameters λ and μ are set to 0.9 and 0.1, respectively, to emphasize the importance of classification accuracy in the optimization process.

## 4. Experiment Results and Discussion

To verify the effectiveness of the proposed HBACO, systematic experiments are conducted in this study. The performance of HBACO and SAMACO is compared with that of several representative algorithms, including basic swarm intelligence methods (ACO, PSO, GA), improved and novel swarm intelligence methods (MSGWO, BQPSO, GOOSE), and deep learning hybrid methods (NPDOA, ACO-RNN, ICVD-ACOEDL). All compared algorithms are implemented in MATLAB R2024a. All experimental results presented in this chapter are obtained on a personal computer equipped with an Intel(R) Core(TM) i5-14600KF CPU, running at 2.3 GHz with 32.0 GB RAM under the Windows 11 operating system.

### 4.1. Dataset

To evaluate the performance of the proposed method, 13 representative medical datasets from the field of bioinformatics are selected, as shown in Table 2. The number of features ranges from 2000 to 22,283, and the number of samples ranges from 50 to 203, showing the typical characteristics of high-dimensional small sample data. Moreover, these datasets all suffer from severe class imbalance, which brings difficulties to feature selection and accurate classification, and provides a basis for verifying the practical application value and optimization performance of the algorithm.

### 4.2. Experimental Settings

To ensure the stable convergence of all compared algorithms, the maximum number of iterations maxIter is set to 100 for all methods, and the population size popSize is set to 10. The weight of classification error λ and the weight of feature selection ratio μ are set to 0.9 and 0.1, respectively, which emphasizes the classification accuracy while considering feature dimension reduction. The parameter *k* of the KNN classifier is set to 5 to balance the bias and variance of the classifier and reduce the influence of random factors as much as possible. Prior to the experiments, all datasets were preprocessed first. To eliminate the impact of feature scales on algorithm search and classification performance, we normalized all features and scaled them to the range of [0, 1]. To ensure a fair evaluation and avoid data leakage, a stratified 5-fold cross-validation strategy was employed in all experiments. In each fold, feature selection was performed exclusively on the training subset, and the obtained feature subset was subsequently applied to the corresponding test subset for classification evaluation. Therefore, the testing data were completely isolated from the feature selection process. In addition, stratified sampling was adopted to preserve the class distribution across different folds, which is particularly important for high-dimensional small-sample datasets. On each dataset, all algorithms are executed independently for 10 times, and the average values are recorded as the final experimental results to guarantee the stability and reliability. The detailed experimental parameters are listed in the Table 3.

### 4.3. Experimental Results and Comparison

To further verify the effectiveness of the proposed HBACO algorithm for high-dimensional small-sample feature selection, comprehensive comparative experiments are conducted in this section. HBACO is compared with SAMACO and nine other state-of-the-art algorithms. The evaluation mainly focuses on classification accuracy, average number of selected features, and running time. The maximum accuracy, minimum accuracy, and standard deviation (STD) are also used to evaluate the optimization ability, dimension reduction performance, stability, and efficiency of HBACO.

#### 4.3.1. Robustness Analysis with Different Classifiers

To further evaluate the robustness and generalization ability of the selected feature subsets, we conduct additional experiments using three representative classifiers, i.e., KNN, SVM, and Random Forest, on five benchmark datasets. The detailed classification results are reported in Table 4.

As shown in the results, the proposed method consistently achieves strong classification performance across different classifiers. Among them, SVM generally yields slightly better performance than KNN and Random Forest on most datasets, indicating that the selected features are particularly effective under margin-based decision boundaries. KNN also demonstrates competitive performance, suggesting that the local structure of the selected feature space is well preserved. Although Random Forest exhibits relatively lower accuracy in some cases, the overall performance remains stable across all datasets.

It is also worth noting that the performance variations across different classifiers are expected due to their distinct learning mechanisms and inductive biases. Despite these differences, the proposed HBACO-based feature selection method maintains consistently high accuracy across all classifiers and datasets, which indicates that the selected feature subsets are not overly dependent on a specific classification model.

Overall, these results demonstrate that the proposed method can produce robust and discriminative feature subsets that generalize well across different classification paradigms, thereby further confirming its effectiveness in high-dimensional small-sample learning scenarios.

#### 4.3.2. Ablation Study

To quantitatively analyze the contribution of each component in HBACO, two groups of ablation experiments are designed. (1) Collaborative framework ablation: compare SAMACO, the two-population version HBACO-2Pop (only including the ACO search group and HRO evolution group, removing the cooperative feedback group and dual-population knowledge sharing), and the complete HBACO. (2) Heuristic strategy ablation: on the basis of the complete framework, remove the self-attention correlation module (HBACO w/o SA) and the generational inheritance increment module (HBACO w/o GI), respectively. All variants are run independently 10 times under the same data split, the same fitness function, and the same classifier settings.

Table 5 shows the overall performance of the framework ablation averaged over 13 datasets.

Among the 13 datasets, 9 datasets show a clear progressive relationship of SAMACO < HBACO-2Pop < HBACO, indicating that the collaborative framework improves both classification accuracy and feature compression performance on difficult high-dimensional datasets. This may be due to the knowledge sharing between ACO and HRO, which guides individuals in the population to find better solutions.

The mean values of the four datasets Lung, Lymphoma, Prostate_GE, and Leukemia_2 show little difference, indicating that the framework gain is limited when the inter-class separability is strong.

Table 6 summarizes the overall performance of heuristic ablation over 13 datasets.

It can be seen from the experimental results that both SA and GI are core contribution modules of HBACO, and removing either module will lead to obvious performance degradation.

After removing the self-attention mechanism (HBACO w/o SA) or the generational inheritance increment (HBACO w/o GI), the average classification accuracy on the 13 datasets decreases. Meanwhile, the average number of feature subsets (AvgN) increases significantly, and the standard deviation rises slightly, indicating that SA and GI play key roles in improving accuracy, compressing features, and enhancing stability.

SA contributes more prominently on ultra-high-dimensional datasets (SMK_CAN_187, GLI_85, CLL_SUB_111), indicating that it can effectively mine feature correlations and suppress redundancy, and is especially suitable for high-dimensional small-sample scenarios. GI leads to a larger performance drop on datasets with complex data distribution and strong dependence on iterative optimization, such as the Leukemia series and Lung, indicating that it guides the search through historical information and significantly improves the global exploration ability of the algorithm.

#### 4.3.3. Comparison with Basic Swarm Intelligence Algorithms

To validate the effectiveness of HBACO, it is compared with SAMACO and three classic swarm intelligence algorithms (ACO, PSO, GA) on 13 high-dimensional small-sample datasets. The optimization ability, dimension reduction performance, and running speed are tested, and the results are listed in Table 7.

In terms of classification accuracy, HBACO achieves the highest mean accuracy on 12 datasets and is slightly better than SAMACO by a margin of 0.04–0.96%. The superiority of HBACO is more obvious on challenging datasets such as SMK_CAN_187 and CLL_SUB_111. On SMK_CAN_187, HBACO improves the mean accuracy to 89.53%, which is 0.75% higher than SAMACO, demonstrating that the inheritance of dominant traits in the hybrid breeding mechanism is beneficial for selecting superior individuals. Only on the Leukemia dataset does GA achieve a slightly higher accuracy (98.17%) than HBACO (97.42%), which may be due to the matching between the dataset characteristics and the global random search strategy of GA.

In terms of dimension reduction, the average number of selected features of HBACO is lower than that of SAMACO on all datasets, with a reduction range of 3–22.5%. On SMK_CAN_187, the number of selected features is reduced from 558.00 (SAMACO) to 432.70 (HBACO), representing a 22.5% reduction. Compared with ACO, PSO, and GA, HBACO shows stronger dimension reduction ability, confirming the effectiveness of ACO and hybrid breeding mechanism in feature selection.

In terms of running time, HBACO is slightly higher than SAMACO by 6.8–13.6% due to the introduction of collaborative screening and information interaction modules. However, it is significantly faster than traditional methods, with a time reduction of 52–78% compared with ACO and 56–82% compared with GA. Even on the highest-dimensional datasets GLI_85 and SMK_CAN_187, HBACO only costs 33.67 s and 35.48 s, indicating that the co-evolutionary framework does not cause obvious efficiency loss.

#### 4.3.4. Comparison with Improved and New Swarm Intelligence Algorithms

To further evaluate the overall performance of HBACO, it is compared with SAMACO, improved swarm intelligence algorithms (MSGWO, BQPSO), and the newly proposed GOOSE algorithm. The results are listed in Table 8.

In terms of classification accuracy, HBACO shows obvious advantages. On the Lung dataset, HBACO achieves 98.82%, which is 0.59% higher than SAMACO, and the maximum accuracy reaches 99.35%, only slightly lower than BQPSO (99.49%). On Leukemia_3, HBACO provides 98.56% mean accuracy, 0.44% higher than SAMACO, and the maximum accuracy is 99.87%, which is better than GOOSE (99.71%). MSGWO and BQPSO exhibit much lower accuracy, with a performance gap of more than 5–8% on datasets such as ALLAML and CLL_SUB_111, indicating that traditional optimization methods cannot effectively identify high-quality feature subsets in high-dimensional space.

In terms of feature reduction, HBACO selects the fewest features on all 13 datasets. On SMK_CAN_187, HBACO selects only 432.70 features, which is 22.5% less than SAMACO, 54.4% less than GOOSE, 81.5% less than BQPSO, and 75.0% less than MSGWO. On GLIOMA, HBACO selects only 42.80 features, 5.3% less than SAMACO, and 76.5–97.6% less than other competitors. This significant dimension reduction is attributed to the dominant trait purification mechanism in HBACO, which retains important interactive features while eliminating redundancy.

In terms of running time, HBACO is superior to MSGWO, BQPSO, and GOOSE. It reduces time consumption by approximately 76–89% compared with MSGWO and 83–92% compared with BQPSO. Although BQPSO can achieve relatively high accuracy on individual datasets, its computational cost is too high to support rapid optimization for large-scale high-dimensional data.

In terms of stability, the STD values of HBACO are lower than those of most competing algorithms. On Lung, the STD of HBACO is 0.26, lower than SAMACO (0.43), BQPSO (0.29), and GOOSE (0.61). GOOSE shows an STD of 3.25 on GLI_85, and MSGWO shows an STD of 1.55 on Leukemia, indicating unstable search performance in high-dimensional space. In contrast, the three-population collaborative structure of HBACO effectively balances exploration and exploitation, leading to stronger robustness.

#### 4.3.5. Comparison with Deep Learning Hybrid Methods

To comprehensively validate the advancement of HBACO, it is compared with three deep learning hybrid methods: NPDOA, ACO-RNN, and ICVD-ACOEDL. The results are listed in Table 9.

In terms of classification accuracy, HBACO outperforms all deep learning hybrid methods on 12 out of 13 datasets. On Leukemia_3, HBACO achieves 98.56%, which is 1.08% higher than the best competing method ICVD-ACOEDL. On SMK_CAN_187, HBACO improves accuracy by 0.75% over SAMACO and 1.00–3.09% over the three deep learning methods, demonstrating strong discriminability on highly redundant datasets. Only on ALLAML does ICVD-ACOEDL show slightly higher accuracy than HBACO.

In terms of feature reduction, HBACO selects far fewer features than all deep learning methods. On GLIOMA, HBACO selects only 42.80 features, which is 5.3% less than SAMACO and 95.5–97.2% less than NPDOA, ACO-RNN, and ICVD-ACOEDL. Such a remarkable dimension reduction benefits from the self-attention mechanism and hybrid breeding strategy, which effectively identify key features and avoid redundancy and overfitting caused by deep learning models.

In terms of running time, HBACO reduces time consumption by 43–76% compared with ACO-RNN, 45–77% compared with ICVD-ACOEDL, and 10–30% compared with NPDOA. The efficiency improvement comes from the fact that HBACO does not rely on training complex neural networks but uses co-evolution and heuristic guidance for efficient feature selection.

In terms of stability, HBACO achieves lower STD values than all deep learning methods. On Leukemia, the STD of HBACO is only 0.67, much lower than 3.34 of NPDOA. Deep learning-based methods are sensitive to random initialization, leading to unstable performance, while HBACO shows strong robustness in high-dimensional scenarios.

In summary, HBACO is a co-evolutionary method developed from SAMACO using the hybrid breeding mechanism. It retains the high efficiency and accuracy of SAMACO while further improving classification performance, robustness, and dimension reduction ability. Comprehensive comparisons in accuracy, feature count, efficiency, and stability validate that HBACO provides significant advantages and is effective and practical for feature selection in high-dimensional sparse datasets.

#### 4.3.6. Graphical Analysis and Discussion

**Figure 2:**
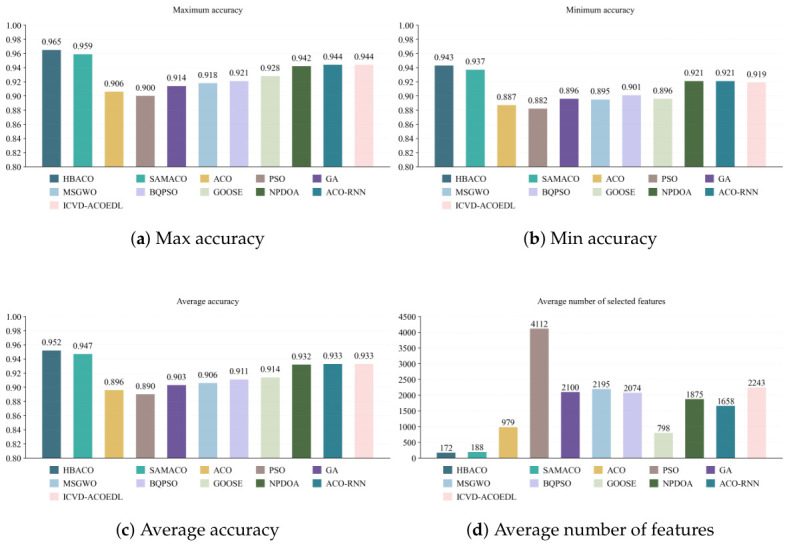
Comprehensive performance comparison of all algorithms on 13 datasets.

Figure 2 presents the overall performance of all algorithms. HBACO achieves the best accuracy, stability, and feature reduction efficiency owing to its three-population collaborative evolution, self-attention feature weighting, and generational inheritance strategy. The cooperative framework enhances global search capability, while attention-based heuristics highlight meaningful features and reduce redundancy. Meanwhile, generational inheritance increment and adaptive pheromone update improve search stability and alleviate premature convergence. Consequently, HBACO maintains higher prediction accuracy with a more compact feature subset, showing superior effectiveness for high-dimensional small-sample feature selection.

**Figure 3:**
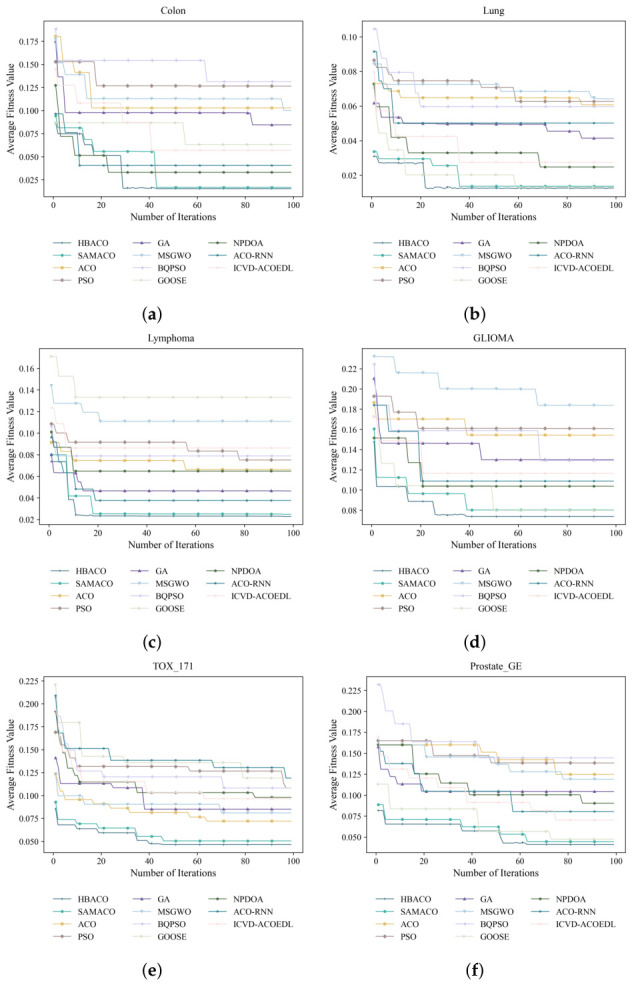

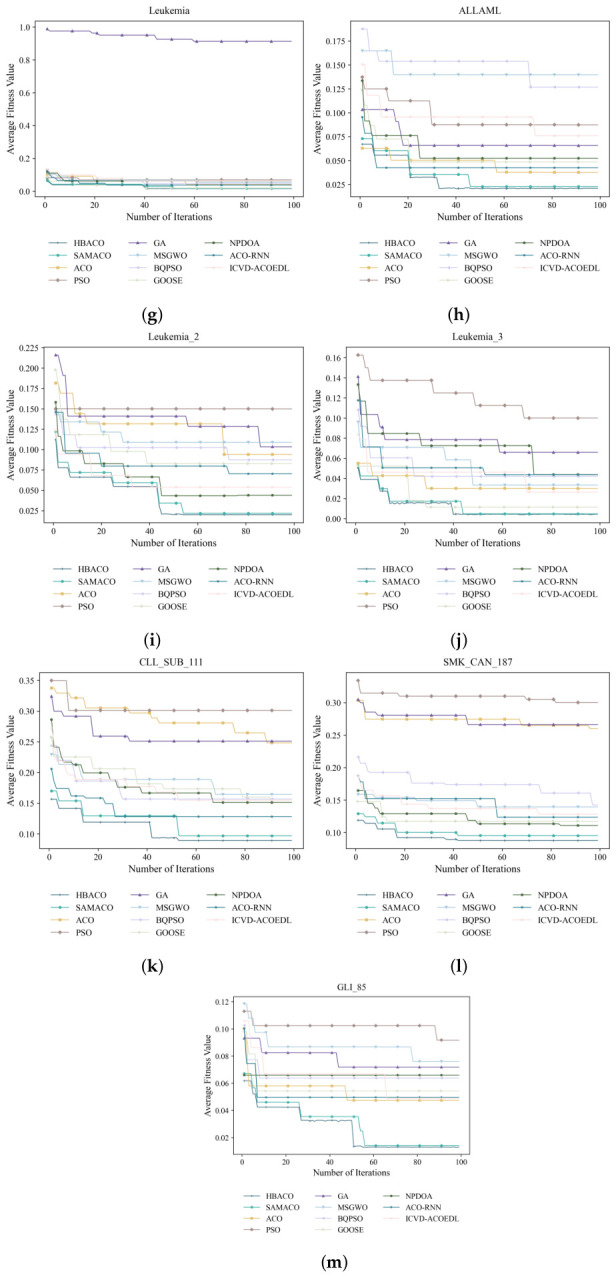
Convergence curves of different datasets: (**a**) Colon dataset; (**b**) Lung dataset; (**c**) Lymphoma dataset; (**d**) GLIOMA dataset; (**e**) TOX_171 dataset; (**f**) Prostate_GE dataset; (**g**) Leukemia dataset; (**h**) ALLAML dataset; (**i**) Leukemia_2 dataset; (**j**) Leukemia_3 dataset; (**k**) CLL_SUB_111 dataset; (**l**) SMK_CAN_187 dataset; (**m**) GLI_85 dataset.

Figure 3 depicts the convergence performance on 13 datasets, where HBACO shows distinctly faster convergence and better stability owing to its three-population collaborative framework, adaptive strategy, and dual-population pheromone update mechanism. It reaches stability within only 30–50 iterations, 5–15 generations earlier than SAMACO and 20–50 generations earlier than traditional and deep learning-based methods, especially on high-dimensional datasets like GLI_85, as heuristic guidance and information sharing effectively reduce redundant exploration and balance exploration and exploitation. While achieving final fitness close to SAMACO, HBACO yields smoother and more stable curves without late fluctuations observed in GOOSE and ICVD-ACOEDL, thanks to the generational inheritance increment that suppresses random oscillations and avoids invalid searches. These results demonstrate that HBACO effectively improves convergence speed and robustness while maintaining high optimization accuracy, making it well-suited for high-dimensional small-sample feature selection.

**Figure 4:**
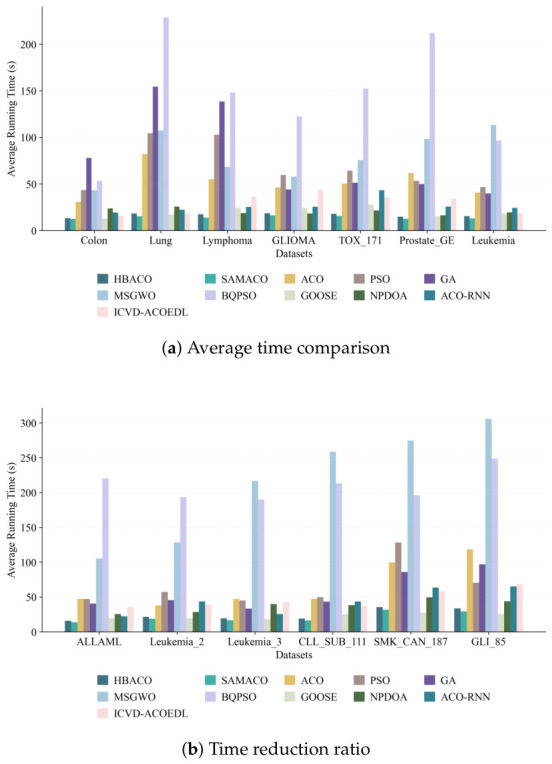
Average time consumption comparison of all algorithms on 13 datasets.

Figure 4 presents the average time consumption of all algorithms on the 13 datasets, highlighting the overall computational efficiency advantage of HBACO. In general, the time cost of HBACO is slightly higher than that of SAMACO, with an increase of 10% to 16%, but it outperforms all other compared algorithms by reducing the time consumption by 30% to 85%. This is mainly because HBACO is an improved version based on the solid foundation of SAMACO. While retaining the main advantages of SAMACO and simplifying the search process, the additional operations introduced by the collaborative feedback group selection and information exchange between the two populations only bring limited extra overhead. Consequently, the total execution time is almost the same as SAMACO and much lower than all other competitors. This demonstrates that HBACO can not only improve classification accuracy and perform efficient feature dimension reduction but also achieve excellent convergence performance, verifying its high efficiency in handling high-dimensional small-sample feature selection tasks.

#### 4.3.7. Nonparametric Statistical Tests

To explore the differences among the proposed HBACO, SAMACO, and other swarm intelligence-based feature selection methods, the Wilcoxon signed-rank test, Friedman test, and Holm post hoc test are adopted for statistical analysis of the experimental results.

The results of the Wilcoxon signed-rank test are presented in Table 10. In the comparison between HBACO and SAMACO, $W^{+}=91$ and $W^{-}=0$, indicating that HBACO outperforms SAMACO in all test cases with extremely high statistical significance. When compared with other algorithms, only the comparisons with GA and ICVD-ACOEDL yield $W^{-}=1$, further highlighting the performance superiority of HBACO.

The results of the Friedman test are given in Table 11. The obtained $p=1.2\times 10^{-15}<0.01$ indicates significant overall differences among all compared algorithms. HBACO achieves an average rank of 1.15 and ranks first, demonstrating the best overall performance. Although SAMACO outperforms subsequent methods such as ICVD-ACOEDL and ACO-RNN, there is a clear gap between SAMACO and HBACO, further verifying that the performance improvement of HBACO over SAMACO is consistent and stable across different cases.

The results of the Holm test are reported in Table 12. The *p*-value for the comparison between HBACO and SAMACO is less than the corresponding α/rank value; therefore, the null hypothesis is rejected, confirming that the performance difference between HBACO and SAMACO is statistically significant. The Holm test results of HBACO against all other algorithms also show that all *p*-values are smaller than their respective α/rank thresholds, leading to rejection of the null hypotheses for all comparisons. This indicates that HBACO performs significantly better than all compared algorithms, which is consistent with the results of the Wilcoxon signed-rank test.

The statistical test results demonstrate that HBACO exhibits significant differences from all compared algorithms, further verifying the uniqueness and superiority of HBACO in terms of performance, and ensuring the reliability and importance of the experimental results.

## 5. Conclusions

This paper proposed HBACO, a hybrid breeding-inspired co-evolutionary ant colony optimization algorithm for high-dimensional small-sample feature selection. Motivated by the biological principles of hybrid breeding, including genetic recombination, advantageous trait inheritance, and population selection, HBACO constructs a three-population collaborative framework consisting of an ACO-based search population, an HRO-based evolutionary population, and a cooperative feedback population. Through the integration of exploration, evolution, and information sharing, the proposed framework enhances population diversity and improves the balance between global exploration and local exploitation. Furthermore, a correlation- and inheritance-guided heuristic strategy together with a dual-population pheromone updating mechanism is introduced to improve feature subset construction and knowledge sharing during the optimization process.

Experimental results on 13 high-dimensional benchmark datasets demonstrate the effectiveness of HBACO. Compared with 10 representative feature selection algorithms, HBACO achieved the best overall performance in terms of classification accuracy, feature reduction capability, and convergence behavior. In particular, HBACO improved the average classification accuracy by 3.9% and achieved an average feature dimensionality reduction rate of 91.4%, while statistical tests further confirmed the significance of the obtained results.

Despite these encouraging results, several limitations remain. The population allocation strategy and several control parameters are currently determined empirically, and the proposed method has not yet been validated on extremely large-scale datasets or more complex learning scenarios.

Future work will focus on extending HBACO to multi-label and imbalanced classification tasks, investigating adaptive population adjustment strategies, and exploring more scalable implementations for large-scale feature selection problems.

## Figures and Tables

**Table 1:** Comparison of representative swarm intelligence-based feature selection methods.

Method	Search Mechanism	Feature Redundancy Handling	Limitation
GA	Genetic Operators	Indirect	Premature convergence
PSO	Velocity Update	Weak	Loss of diversity
ACO	Pheromone Search	Moderate	Local optima
SAMACO	Attention-guided ACO	Strong	Limited information interaction
HRO	Hybrid Breeding Evolution	Moderate	Slower convergence rate

**Table 2:** Dataset description.

Dataset	Features	Samples	Classes
Colon	2000	62	2
Lung	3312	203	5
Lymphoma	4026	96	9
GLIOMA	4434	50	4
TOX_171	5748	171	4
Prostate_GE	5966	102	2
Leukemia	7070	72	2
ALLAML	7129	72	2
Leukemia_2	7129	72	4
Leukemia_3	11,225	72	3
CLL_SUB_111	11,340	111	3
SMK_CAN_187	19,993	187	2
GLI_85	22,283	85	2

**Table 3:** Parameter settings.

Symbol	Value
α	1
β	4
λ	0.01
$\theta_{\min}$	0.05
$\theta_{\max}$	0.3
$d_{0}$	500
$\gamma_{\min}$	0.6
$\gamma_{\max}$	1.0
SC	8

**Table 4:** Classification results with different classifiers (%).

Dataset	KNN	SVM	Random Forest
Colon	97.31 ± 1.27	98.04 ± 0.93	95.82 ± 2.11
Lung	98.82 ± 0.26	99.12 ± 0.18	98.47 ± 0.41
Leukemia_3	98.56 ± 0.81	98.73 ± 0.69	97.94 ± 1.12
SMK_CAN_187	89.53 ± 0.42	90.14 ± 0.37	88.21 ± 0.93
CLL_SUB_111	88.27 ± 1.36	89.02 ± 1.11	86.75 ± 2.04

**Table 5:** Overall performance of framework ablation averaged over 13 datasets.

Metric	SAMACO	HBACO-2Pop	HBACO
Average Mean (%)	94.65	96.50	96.83
Average AvgN	244.18	232.86	221.35
Average STD	0.89	0.84	0.79

**Table 6:** Overall performance of heuristic ablation averaged over 13 datasets.

Metric	HBACO w/o SA	HBACO w/o GI	HBACO
Average Mean (%)	95.80	95.76	96.83
Average AvgN	234.62	236.18	221.35
Average STD	0.86	0.88	0.79

**Table 7:** Comparison between the proposed method and basic swarm intelligence algorithms.

Datasets	Algorithm	Max	Min	Mean + STD	AvgN	Time
Colon	HBACO	98.83	96.13	97.31 + 1.27	65.50	13.27
	SAMACO	98.52	95.41	96.45 + 1.18	68.20	12.43
	ACO	93.59	90.26	92.04 + 1.42	902.10	30.72
	PSO	91.48	86.65	88.58 + 1.56	995.40	43.33
	GA	93.32	90.10	91.06 + 1.47	488.00	77.86
Lung	HBACO	99.35	98.11	98.82 + 0.26	143.80	18.36
	SAMACO	99.01	97.68	98.23 + 0.43	157.60	15.20
	ACO	97.52	96.05	97.08 + 0.49	1266.20	82.15
	PSO	97.54	96.55	97.24 + 0.32	1341.00	104.48
	GA	98.52	97.42	98.03 + 0.15	931.00	154.41
Lymphoma	HBACO	97.85	96.24	96.71 + 0.31	188.30	17.36
	SAMACO	97.79	96.24	96.67 + 0.35	195.10	13.93
	ACO	95.96	94.92	95.12 + 0.51	1201.40	54.95
	PSO	96.13	95.54	95.92 + 0.21	1621.20	102.88
	GA	97.00	95.96	96.17 + 0.43	782.40	138.44
GLIOMA	HBACO	92.50	90.12	91.36 + 0.85	42.80	18.52
	SAMACO	91.20	89.50	90.80 + 0.92	45.20	16.28
	ACO	86.00	84.00	84.80 + 1.19	1254.30	46.31
	PSO	86.00	84.00	84.50 + 1.25	1529.80	59.51
	GA	88.20	86.30	87.60 + 1.08	1050.80	43.93
TOX_171	HBACO	95.12	92.87	93.68 + 0.89	78.60	17.95
	SAMACO	94.55	92.06	93.01 + 0.96	82.30	15.64
	ACO	92.74	90.40	91.80 + 1.05	387.00	50.21
	PSO	92.94	91.43	92.22 + 0.81	2563.80	64.13
	GA	93.91	92.15	92.79 + 0.73	1741.60	51.31
Prostate_GE	HBACO	95.87	93.72	94.93 + 0.68	55.30	14.82
	SAMACO	95.16	93.08	94.56 + 0.75	58.60	12.66
	ACO	90.42	87.25	88.95 + 1.46	2298.20	61.67
	PSO	89.22	88.24	88.82 + 0.65	2465.30	53.37
	GA	91.18	89.22	90.39 + 0.83	1492.20	49.77
Leukemia	HBACO	98.65	96.83	97.42 + 0.67	92.80	15.39
	SAMACO	98.22	96.29	96.78 + 0.73	96.50	13.07
	ACO	97.52	96.45	97.16 + 0.47	1354.80	40.88
	PSO	97.81	96.22	97.35 + 0.59	3485.40	46.67
	GA	100.00	97.61	98.17 + 0.85	1540.00	39.89
ALLAML	HBACO	98.24	95.76	96.91 + 1.13	129.50	15.78
	SAMACO	97.67	95.03	96.34 + 1.25	135.00	13.55
	ACO	96.37	95.63	95.87 + 0.57	356.20	47.39
	PSO	95.83	94.44	95.00 + 0.64	3551.80	46.92
	GA	95.83	94.44	95.06 + 0.75	2009.80	40.63
Leukemia_2	HBACO	98.31	96.75	97.46 + 0.38	83.20	21.45
	SAMACO	97.72	96.22	96.93 + 0.43	87.50	18.90
	ACO	90.28	88.89	89.72 + 0.53	465.00	37.76
	PSO	88.89	88.89	88.89 + 0.00	3555.60	57.28
	GA	91.67	88.89	90.00 + 1.46	2022.60	45.40
Leukemia_3	HBACO	99.87	97.92	98.56 + 0.81	135.70	19.26
	SAMACO	99.61	97.38	98.12 + 0.93	141.20	16.54
	ACO	97.22	94.44	95.56 + 1.29	561.00	47.02
	PSO	94.44	93.06	93.61 + 0.67	5611.40	44.89
	GA	95.83	94.44	94.72 + 0.62	3194.80	33.41
CLL_SUB_111	HBACO	90.15	87.03	88.27 + 1.36	142.80	18.93
	SAMACO	89.37	86.19	87.56 + 1.52	149.00	16.48
	ACO	72.97	72.07	72.43 + 0.36	567.00	47.44
	PSO	72.07	68.47	69.55 + 1.72	5626.20	49.62
	GA	74.77	72.07	73.15 + 1.06	2720.20	43.27
SMK_CAN_187	HBACO	90.58	88.94	89.53 + 0.42	432.70	35.48
	SAMACO	89.72	88.13	88.78 + 0.49	558.00	31.74
	ACO	71.66	70.05	70.70 + 0.69	1000.00	99.68
	PSO	72.19	70.59	71.02 + 0.73	9970.60	128.01
	GA	73.26	71.66	72.41 + 0.86	5102.20	85.79
GLI_85	HBACO	99.23	95.87	96.85 + 1.07	641.30	33.67
	SAMACO	98.76	95.18	96.23 + 1.15	668.60	29.34
	ACO	95.29	92.94	93.88 + 0.95	1114.00	118.27
	PSO	95.37	93.01	94.05 + 0.82	11,134.20	70.40
	GA	94.12	94.12	94.12 + 0.00	4224.20	96.99

**Table 8:** Results of comparison with improved and new swarm intelligence algorithms.

Datasets	Algorithm	Max	Min	Mean + STD	AvgN	Time
Colon	HBACO	98.83	96.13	97.31 + 1.27	65.50	13.27
	SAMACO	98.52	95.41	96.45 + 1.18	68.20	12.43
	MSGWO	93.46	91.92	92.83 + 0.76	825.00	43.25
	BQPSO	88.97	88.97	88.97 + 0.00	639.00	53.27
	GOOSE	93.90	88.48	91.34 + 1.64	166.40	12.85
Lung	HBACO	99.35	98.11	98.82 + 0.26	143.80	18.36
	SAMACO	99.01	97.68	98.23 + 0.43	157.60	15.20
	MSGWO	97.52	95.86	96.67 + 0.49	1384.20	107.31
	BQPSO	99.49	97.97	98.52 + 0.29	1527.40	228.63
	GOOSE	99.49	97.56	98.42 + 0.61	281.80	16.97
Lymphoma	HBACO	97.85	96.24	96.71 + 0.31	188.30	17.36
	SAMACO	97.79	96.24	96.67 + 0.35	195.10	13.93
	MSGWO	92.68	91.85	92.52 + 0.41	1814.00	68.23
	BQPSO	94.75	93.97	94.25 + 0.39	1271.60	148.32
	GOOSE	86.41	81.08	84.34 + 2.05	439.40	24.32
GLIOMA	HBACO	92.50	90.12	91.36 + 0.85	42.80	18.52
	SAMACO	91.20	89.50	90.80 + 0.92	45.20	16.28
	MSGWO	84.00	82.00	82.40 + 0.84	1765.50	57.96
	BQPSO	90.05	84.41	86.08 + 2.78	1759.70	122.37
	GOOSE	91.59	90.39	91.18 + 0.39	182.20	24.40
TOX_171	HBACO	95.12	92.87	93.68 + 0.89	78.60	17.95
	SAMACO	94.55	92.06	93.01 + 0.96	82.30	15.64
	MSGWO	94.72	91.43	92.77 + 1.52	1927.10	75.62
	BQPSO	91.39	88.27	90.30 + 1.72	1762.30	152.33
	GOOSE	87.68	85.37	86.22 + 0.85	470.40	27.83
Prostate_GE	HBACO	95.87	93.72	94.93 + 0.68	55.30	14.82
	SAMACO	95.16	93.08	94.56 + 0.75	58.60	12.66
	MSGWO	90.23	87.10	88.36 + 0.83	1855.30	98.20
	BQPSO	87.43	86.48	86.93 + 0.43	1877.30	211.93
	GOOSE	94.12	90.98	92.70 + 0.95	1283.00	15.55
Leukemia	HBACO	98.65	96.83	97.42 + 0.67	92.80	15.39
	SAMACO	98.22	96.29	96.78 + 0.73	96.50	13.07
	MSGWO	97.53	94.82	95.91 + 1.55	2281.60	113.28
	BQPSO	98.04	95.39	96.81 + 1.17	1973.00	96.52
	GOOSE	98.52	96.55	96.93 + 0.97	454.60	18.30
ALLAML	HBACO	98.24	95.76	96.91 + 1.13	129.50	15.78
	SAMACO	97.67	95.03	96.34 + 1.25	135.00	13.55
	MSGWO	88.76	86.19	87.52 + 0.82	2755.20	105.33
	BQPSO	90.00	88.12	89.18 + 0.39	2625.30	220.18
	GOOSE	96.06	94.37	95.43 + 0.63	926.20	19.50
Leukemia_2	HBACO	98.31	96.75	97.46 + 0.38	83.20	21.45
	SAMACO	97.72	96.22	96.93 + 0.43	87.50	18.90
	MSGWO	93.40	90.31	92.18 + 1.32	3521.80	128.37
	BQPSO	94.55	92.37	93.73 + 0.82	2721.50	193.16
	GOOSE	92.16	89.10	90.42 + 1.29	867.20	19.36
Leukemia_3	HBACO	99.87	97.92	98.56 + 0.81	135.70	19.26
	SAMACO	99.61	97.38	98.12 + 0.93	141.20	16.54
	MSGWO	98.85	96.62	97.18 + 0.85	2585.80	216.33
	BQPSO	97.18	96.35	96.88 + 0.43	1872.10	189.74
	GOOSE	99.71	96.26	97.22 + 0.91	995.00	18.14
CLL_SUB_111	HBACO	90.15	87.03	88.27 + 1.36	142.80	18.93
	SAMACO	89.37	86.19	87.56 + 1.52	149.00	16.48
	MSGWO	83.27	80.20	81.93 + 1.22	1566.30	258.44
	BQPSO	85.39	82.73	84.28 + 1.36	2872.40	212.85
	GOOSE	84.44	82.03	83.77 + 1.08	1264.60	25.31
SMK_CAN_187	HBACO	90.58	88.94	89.53 + 0.42	432.70	35.48
	SAMACO	89.72	88.13	88.78 + 0.49	558.00	31.74
	MSGWO	85.47	83.71	84.56 + 0.94	1729.00	274.63
	BQPSO	85.47	82.39	84.28 + 1.07	2338.10	195.84
	GOOSE	87.49	84.98	86.62 + 1.27	949.60	27.57
GLI_85	HBACO	99.23	95.87	96.85 + 1.07	641.30	33.67
	SAMACO	98.76	95.18	96.23 + 1.15	668.60	29.34
	MSGWO	93.81	91.28	92.74 + 0.96	4528.60	305.80
	BQPSO	94.77	93.81	94.25 + 0.36	3722.30	248.32
	GOOSE	95.01	87.97	93.61 + 3.25	2097.00	25.73

**Table 9:** Comparison between the proposed method and deep learning hybrid methods.

Datasets	Algorithm	Max	Min	Mean + STD	AvgN	Time
Colon	HBACO	98.83	96.13	97.31 + 1.27	65.50	13.27
	SAMACO	98.52	95.41	96.45 + 1.18	68.20	12.43
	NPDOA	97.63	95.48	96.31 + 0.85	235.60	23.65
	ACO-RNN	97.93	94.38	96.54 + 1.34	437.20	19.25
	ICVD-ACOEDL	95.44	92.72	94.18 + 1.29	318.60	15.80
Lung	HBACO	99.35	98.11	98.82 + 0.26	143.80	18.36
	SAMACO	99.01	97.68	98.23 + 0.43	157.60	15.20
	NPDOA	99.13	96.85	98.46 + 1.21	561.20	25.63
	ACO-RNN	97.35	94.82	96.34 + 1.26	871.20	22.38
	ICVD-ACOEDL	98.71	95.48	97.19 + 1.05	526.10	18.33
Lymphoma	HBACO	97.85	96.24	96.71 + 0.31	188.30	17.36
	SAMACO	97.79	96.24	96.67 + 0.35	195.10	13.93
	NPDOA	96.37	94.28	95.66 + 0.92	1294.20	18.74
	ACO-RNN	97.93	95.47	96.52 + 0.83	763.60	25.38
	ICVD-ACOEDL	94.39	92.58	93.63 + 1.25	1438.40	36.12
GLIOMA	HBACO	92.50	90.12	91.36 + 0.85	42.80	18.52
	SAMACO	91.20	89.50	90.80 + 0.92	45.20	16.28
	NPDOA	90.88	88.79	89.65 + 0.81	961.40	18.22
	ACO-RNN	91.08	88.39	90.25 + 1.48	1266.20	25.47
	ICVD-ACOEDL	90.88	88.21	89.72 + 1.21	1527.20	43.62
TOX_171	HBACO	95.12	92.87	93.68 + 0.89	78.60	17.95
	SAMACO	94.55	92.06	93.01 + 0.96	82.30	15.64
	NPDOA	91.60	91.17	91.43 + 0.27	1288.20	21.44
	ACO-RNN	90.36	85.39	87.92 + 3.18	1847.60	43.20
	ICVD-ACOEDL	92.34	88.73	90.62 + 2.75	1582.40	35.48
Prostate_GE	HBACO	95.87	93.72	94.93 + 0.68	55.30	14.82
	SAMACO	95.16	93.08	94.56 + 0.75	58.60	12.66
	NPDOA	90.61	90.45	90.54 + 0.13	352.60	16.18
	ACO-RNN	92.85	91.76	92.24 + 0.52	962.30	25.71
	ICVD-ACOEDL	94.87	92.65	93.85 + 1.28	1436.20	34.28
Leukemia	HBACO	98.65	96.83	97.42 + 0.67	92.80	15.39
	SAMACO	98.22	96.29	96.78 + 0.73	96.50	13.07
	NPDOA	95.26	89.76	91.99 + 3.34	1386.60	19.54
	ACO-RNN	97.31	96.29	96.95 + 0.48	952.60	24.36
	ICVD-ACOEDL	95.49	92.43	94.18 + 1.35	1585.20	18.37
ALLAML	HBACO	98.24	95.76	96.91 + 1.13	129.50	15.78
	SAMACO	97.67	95.03	96.34 + 1.25	135.00	13.55
	NPDOA	97.88	94.93	96.55 + 1.57	2375.00	25.63
	ACO-RNN	97.67	94.93	96.28 + 1.22	1528.30	22.36
	ICVD-ACOEDL	98.36	96.31	97.25 + 0.92	4362.20	35.28
Leukemia_2	HBACO	98.31	96.75	97.46 + 0.38	83.20	21.45
	SAMACO	97.72	96.22	96.93 + 0.43	87.50	18.90
	NPDOA	97.36	94.58	96.11 + 1.22	1429.30	28.35
	ACO-RNN	96.22	94.14	95.25 + 0.58	2581.10	43.52
	ICVD-ACOEDL	97.36	95.41	96.57 + 0.86	2136.20	38.60
Leukemia_3	HBACO	99.87	97.92	98.56 + 0.81	135.70	19.26
	SAMACO	99.61	97.38	98.12 + 0.93	141.20	16.54
	NPDOA	97.48	96.36	96.95 + 0.47	2382.40	39.77
	ACO-RNN	97.38	97.38	97.38 + 0.00	2258.30	25.63
	ICVD-ACOEDL	98.63	96.25	97.48 + 0.88	1577.30	42.63
CLL_SUB_111	HBACO	90.15	87.03	88.27 + 1.36	142.80	18.93
	SAMACO	89.37	86.19	87.56 + 1.52	149.00	16.48
	NPDOA	85.72	83.44	84.57 + 0.93	2573.40	38.07
	ACO-RNN	87.48	85.69	86.35 + 0.94	1752.30	43.65
	ICVD-ACOEDL	85.72	84.62	85.10 + 0.36	2981.20	37.30
SMK_CAN_187	HBACO	90.58	88.94	89.53 + 0.42	432.70	35.48
	SAMACO	89.72	88.13	88.78 + 0.49	558.00	31.74
	NPDOA	89.75	87.82	88.53 + 0.85	3727.80	49.58
	ACO-RNN	87.69	84.28	86.44 + 1.47	2577.63	63.57
	ICVD-ACOEDL	88.13	85.25	87.31 + 1.52	4255.20	58.21
GLI_85	HBACO	99.23	95.87	96.85 + 1.07	641.30	33.67
	SAMACO	98.76	95.18	96.23 + 1.15	668.60	29.34
	NPDOA	95.57	93.94	94.68 + 0.81	5813.40	43.69
	ACO-RNN	96.36	93.94	94.95 + 1.22	3758.10	65.21
	ICVD-ACOEDL	97.31	94.28	95.97 + 1.27	5433.20	68.30

**Table 10:** Results of the Wilcoxon signed-rank test.

Algorithm	$W^{+}$	$W^{-}$	*Z*	*p*
SAMACO	91	0	3.7	0.0002
ACO	91	0	3.7	0.0002
PSO	91	0	3.7	0.0002
GA	90	1	3.62	0.0003
MSGWO	91	0	3.7	0.0002
BQPSO	91	0	3.7	0.0002
GOOSE	91	0	3.7	0.0002
NPDOA	91	0	3.7	0.0002
ACO-RNN	91	0	3.7	0.0002
ICVD-ACOEDL	90	1	3.62	0.0003

**Table 11:** Results of the Friedman test.

Algorithm	Average Rank	Final Rank	*p*
HBACO	1.15	1	$1.2\times 10^{-15}<0.01$
SAMACO	3.00	2	
ICVD-ACOEDL	5.08	3	
ACO-RNN	5.15	4	
NPDOA	5.38	5	
GOOSE	6.77	6	
GA	6.85	7	
BQPSO	7.62	8	
ACO	8.08	9	
MSGWO	8.46	10	
PSO	8.46	10	

**Table 12:** Results of the Holm test for HBACO (α=0.05).

Algorithm	*p*	α/Rank	Hypothesis
SAMACO	0.0002	0.0050	Rejected
ACO	0.0002	0.0167	Rejected
PSO	0.0002	0.0250	Rejected
GA	0.0003	0.0100	Rejected
MSGWO	0.0002	0.0500	Rejected
BQPSO	0.0002	0.0125	Rejected
GOOSE	0.0002	0.0071	Rejected
NPDOA	0.0002	0.0083	Rejected
ACO-RNN	0.0002	0.0056	Rejected
ICVD-ACOEDL	0.0003	0.0063	Rejected

## Data Availability

The datasets used in this paper can be accessed via the following link: https://archive.ics.uci.edu/ accessed on 1 June 2025. UCI Machine Learning Repository.
